# Supplementary material for: Decreased Levels of GSH Are Associated with Platinum Resistance in High-Grade Serous Ovarian Cancer
Source: Antioxidants (Basel). 2022 Aug 10;11(8):1544. doi: 10.3390/antiox11081544 (PMC9404763; doi:10.3390/antiox11081544)
Supplement: Supplementary file 1 [file antioxidants-11-01544-s001.zip › antioxidants-1865998-supplementary.pdf]

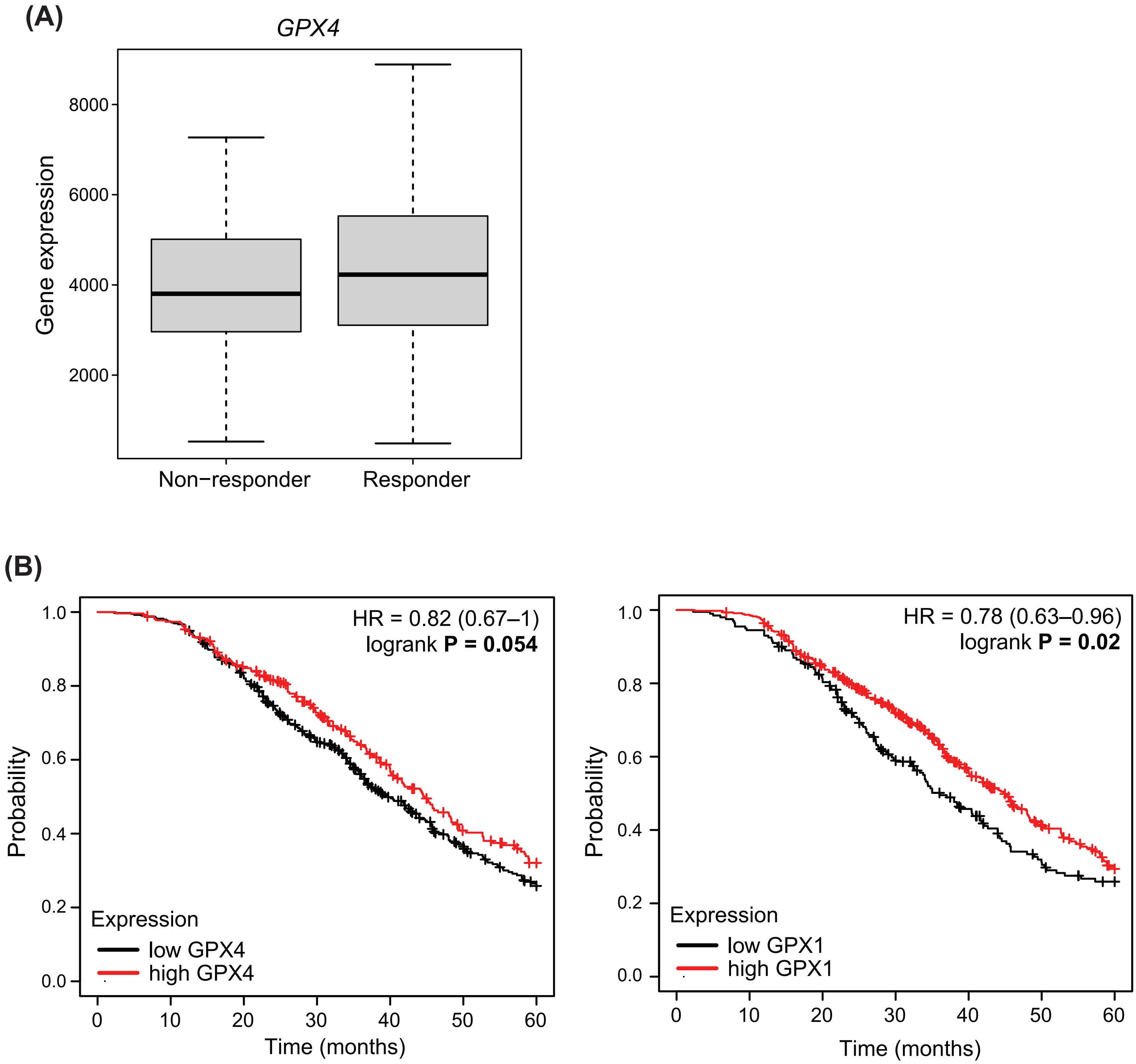

FigureS1. Antioxidant enzymes correlate with response to therapy and survival in HGSOC patients. **A**) Expression levels of GPX4 in HGSOC patients, grouped for their pathological response to platinum-based therapy (complete response vs residual disease after completing the therapy). Statistical significance of differences in gene expression levels in responders (n=461) and non-responders (n=115) was evaluated by the Mann-Whitney test. **B**) Kaplan-Meier estimates of survival obtained on Kaplan Meier plotter analyzing post-progression survival in HGSOC patients treated with platinum-based chemotherapy.
